# GC-IMS identification of early-warning biomarkers and fungal community dynamics during cigar tobacco mold process

**DOI:** 10.3389/fmicb.2025.1595849

**Published:** 2025-07-16

**Authors:** Ge Zhang, Kuo Huang, Qiuxuan Xie, Qingchang Li, Dong Li, Changwen Ye, Chen He, Hongru Xi, Wei Ding, Qingyuan Hu

**Affiliations:** ^1^Center of China Tobacco Standardization, Zhengzhou Tobacco Research Institute of CNTC, Zhengzhou, China; ^2^College of Plant Protection, Southwest University, Chongqing, China; ^3^College of Food Science and Technology, Huazhong Agricultural University, Wuhan, China

**Keywords:** cigar tobacco, GC-IMS, early-warning biomarkers, fungal community, *Aspergillus*, volatile organic compounds

## Abstract

**Introduction:**

Mold-derived contamination in cigar tobacco leaves causes severe economic losses and health risks due to mycotoxin production. This study aimed to identify early-warning biomarkers for mold and elucidate their interaction with fungal communities.

**Method:**

Gas chromatography-ion mobility spectrometry (GC-IMS) combined with high-throughput sequencing was employed to profile volatile organic compounds (VOCs) and fungal communities during artificial molding.

**Results:**

A total of 72 VOCs were detected, with four compounds (2-methyl-1-butanol-M, 2-methyl-1-butanol-D, 2-propanone, and 1-penten-3-ol) identified as early-warning biomarkers through VIP > 1 and *P* < 0.05, showing 1.31.5-fold increases in early mold stages (MB3). Furthermore, fungal diversity sharply declined post-molding (OTUs reduced by 85.7%), with Aspergillus dominating (>99.45% abundance), and exhibiting strong positive correlations with 1-penten-3-ol (ρ = 0.61) and benzaldehyde-M (ρ = 0.67).

**Discussion:**

These findings provide actionable biomarkers for industrial mold prevention and insights into fungal-VOC interaction, with implications for perishable crop storage.

## 1 Introduction

Mold contamination in agricultural products poses severe economic and health threats. Such contamination not only compromises the appearance and sensory quality but also promotes the biosynthesis of hazardous metabolites, including mycotoxins (Cao et al., 2024; Zhou et al., 2024b). In China alone, tobacco mold results in annual losses exceeding 7 billion CNY, while mycotoxins from spoiled leaves endanger consumer safety (Jiabao et al., 2022; Pauly and Paszkiewicz, 2011). Consequently, the early and rapid detection of leaf-borne spoilage fungi is imperative to prevent these contaminants from entering the final product. In addition, studying early-warning biomarkers could help companies develop more appropriate storage strategies to reduce mold growth.

Mold contamination exhibits dynamic progression characteristics, with its risk level transitioning from an initial safe state to subsequent stages of microbial proliferation and mycotoxin production. The process of mold infestation varies widely, encompassing a spectrum from a risk-free state to the emergence of microorganisms and eventual toxin generation. Mold monitoring can be achieved through mycelium observation, characteristic biomarkers, and toxin detection (Gong et al., 2024). Conventional mycelium examination methods face limitations in early warning capabilities due to their time-consuming and observational lag. Consequently, the detection of early-stage biomarkers has emerged as a research priority for achieving timely mold contamination alerts. Notably, mold colonies release VOCs during early metabolic stages, a phenomenon that precedes visible mycelial growth or sporulation by a considerable temporal margin. Furthermore, modern analytical technologies offer enhanced sensitivity, enabling not only more convenient detection but also improved accuracy and precision in VOC quantification. Research efforts to identify early-warning biomarkers through VOCs analysis have been conducted in several fields. For example, 1-octen-3-ol and 3-octanone have been reported to serve as early-warning biomarkers for molds in grain (Hamow et al., 2021; Tian et al., 2023; Zhang et al., 2022). Additionally, some organic acids, aldehydes, and ketones also have been identified as early-warning biomarkers for mold in foods (Afsah-Hejri et al., 2023; Najmeh et al., 2022; Yin et al., 2023).

Advanced detection technologies for VOCs encompass a diverse array of analytical approaches, including electronic nose (E-nose), solid-phase microextraction coupled with gas chromatography-mass spectrometry (SPME-GC-MS), and gas chromatography-ion mobility spectrometry (GC-IMS). For example, E-nose platform was used to detect the VOCs in rice, and established a system to detect the early stages of rice molds (Zhang et al., 2022). Karlshoj et al. (2007) also identified alcohols and ketones as characteristic metabolites from different molds using E-nose. Afsah-Hejri et al. (2023) conducted a comprehensive analysis of *A. flavus*-contaminated pistachios using SPME-GC-MS. Their investigation identified α, β-dimethyl benzenepropanoic acid as a definitive biomarker for *A. flavus* infection in pistachio kernels. Emerging research in tobacco mold contamination has demonstrated the successful application of GC-IMS for identifying specific volatile biomarkers. Such Yu et al. (2023) identified 1-octene-3-alcohol, 1-pentanol, and pentanal as early-stage biomarkers in cigar tobacco leaves following infection by two strains of fungi (*Aspergillus flavus* and *Penicillium chrysogenum*). Furthermore, Wei et al. (2025) characterized dynamic VOC profiles during mold development in cigar components, revealing distinct compound patterns between wrapper and filler leaves. The wrapper exhibited elevated levels of 3-phenyl-2-propen-1-ol, cyclopentanone, 3-methyl-1-butanol, (Z)-3-hexenol, and 4-methoxybenzyl formate, while the filler showed increased 1-pentanol-M, 3-methyl-1-butanol, 2-methyl-1-propanol-M, and 2-propenyl heptanoate concentrations during spoilage. Notably, these successful precedents establish a methodological foundation for employing GC-IMS technology in investigating VOC profiles during tobacco fungal deterioration.

However, VOC emission patterns during mold contamination demonstrate intrinsic connections with microbial metabolic activities. Such as Li et al. (2021a) demonstrated that ethyl acetate-D and 3-hydroxybutan-2-one-D show strong correlations with *Aspergillus flavus* contamination in maize kernels. Nevertheless, fungal spoilage constitutes an ecological succession process rather than singular microbial action, characterized by dynamic microbial community restructuring. Contemporary metagenomic analyses reveal significant α-diversity reductions in phyllosphere microbiota following tobacco mold outbreaks (Fu et al., 2024b; Wei et al., 2024). Particularly, *Aspergillus* was a fungal species associated with a high percentage of moldy tobacco leaves (Wei et al., 2024; Wu et al., 2024a,b; Zhou et al., 2021a). Nevertheless, the causal relationships between VOC flux dynamics and microbial consortia evolution remain poorly elucidated. Therefore, studying VOCs and fungal communities during mold growth, as well as analyzing the correlation between characteristic substances and major fungi not only helps to find early-warning biomarkers but also has deeper significance in revealing the relationship between characteristic substances and microorganisms.

In this study, we subjected cigar tobacco leaves to varying durations of artificial molding under controlled laboratory conditions. The VOCs were systematically analyzed using GC-IMS, revealing statistically significant differential compounds throughout the molding process, particularly identifying early-warning biomarkers during the initial molding phase. Meanwhile, we performed comprehensive analysis of fungal community dynamics on tobacco leaf surfaces through high-throughput sequencing. Furthermore, we established correlation networks between these significantly differentiated compounds and fungal populations using Spearman's correlation analysis. This study elucidates the characteristic VOC profiles associated with molding in cigar tobacco leaves, with particular emphasis on early-stage biomarkers. The findings provide theoretical foundations for developing mold early-warning systems in tobacco processing. Moreover, the identified correlations between characteristic VOCs and predominant fungal species offer valuable insights into microbial contributions to volatile compound formation, potentially guiding targeted mold prevention strategies in tobacco production. Additionally, this study provides methodological guidance for the research of early-warning biomarkers in food preservation, grain storage, and related fields.

## 2 Materials and methods

### 2.1 Reagents and instruments

2-butanone, 2-pentanone, 2-hexanone, 2-heptanone, 2-octanone, and 2-nonanone (Analytical Reagent, 99.999%, Aladdin) were used as reference standards. Ion mobility spectrometry was performed using FlavourSpec^®^ (G.A.S., Germany, Dortmund). CTC-PAL 3 static headspace automatic injection system (CTC Analytics AG, Switzerland) were used.

### 2.2 Preparation and sampling of cigar tobacco leaves

Sterile water was sprayed on the surface of the cigar tobacco leaves, which were collected from the Hainan province until the moisture content reached 30%. The leaves were sealed and placed in a constant temperature and humidity incubator at 28°C and 85% RH. Samples were collected at 3, 7, 10, and 15 days and labeled MB3, MB7, MB10, and MB15, respectively (Yu et al., 2023; Wu et al., 2024b; Wei et al., 2025). The untreated leaves were designated as MB0. The samples were stored at −80°C for subsequent microbial diversity and volatile substance analysis.

### 2.3 DNA extraction and ITS application sequencing

Genomic DNA was extracted using the E.Z.N.A.^®^ soil DNA kit (Omega Bio-tek, Norcross, GA, U.S.), according to the manufacturer's instructions and detected using 1% agarose gel electrophoresis. Amplification was performed using the primers ITS1F 5′3′ CTTGGTCATTTAGAGGAAGTAA) and ITS2R 5′3′ GCTGCGTTCTTCATCGATGC) (Wang et al., 2022), followed by detection of DNA concentration and purity using a NanoDrop 2000 spectrophotometer (Thermo Scientific). Purified PCR products were sequenced on an Illumina NextSeq 2000 platform (Illumina, San Diego, USA) according to the protocols of Majorbio Bio-Pharm Technology Co. Ltd. (Shanghai, China). Each sample was treated in triplicate. Sequencing reads were submitted to the NCBI Sequence Read Archive (SRA) database (Accession Number: PRJNA1215953).

### 2.4 Analysis of volatile organic compounds using GC-IMS

#### 2.4.1 Samples pretreatment

Tobacco samples (1.0 g) were placed in a headspace sampling vial and sealed with a magnetic cap and silicone septum. Then, incubating samples at 80°C for 15 min with the speed of 500 r/min (Yu et al., 2023). Each sample was tested in triplicate.

#### 2.4.2 GC-IMS analysis

##### 2.4.2.1 GC conditions

MXT-WAX column (15 m × 0.53 mm, 1.0 um, Restek, USA); column temperature 60°C; carrier gas: nitrogen (purity ≥ 99.999%). The procedure was as follow: 0–2 min 2.0 mL/min, 2–8 min increase linearly to 10.0 mL/min, 8–10 min increase linearly to 100.0 mL/min, hold for 10 min. The injection temperature was maintained at 80°C.

##### 2.4.2.2 IMS conditions

Iionization source, tritium source (3H); drift tube length, 98 mm; electric field strength, 500 V/cm; drift tube temperature, 45°C; drift gas, nitrogen (purity ≥ 99.999%); and flow rate, 150.0 mL/min. Positive ion mode.

### 2.5 Data processing

Majorbio's platform (https://www.majorbio.com/web/www/index) was used for ITS sequencing data analysis (Ren et al., 2022). The sequences were normalized based on the minimum sequence, excluding sequences related to mitochondria and chloroplasts for operational taxonomic units (OTU) clusters with 97% similarity. The OTU cluster was annotated using the RDP Classifier version 2.11 in Unite (Release 8.0 http://unite.ut.ee/index.php). Alpha diversities, including obs, shannon, simpson, ace, chao, and average indices, were analyzed at the OTU level. The similarity among the microbial communities in different samples was determined by principal coordinate analysis (PCoA) based on unweighted unifrac using R language (v3.3.1). Analysis of similarities (ANOSIM) was used to test the differences in similarities among groups of samples with 999 permutations (Somerfield et al., 2021). The correlation between the top 10 genera was analyzed using Networkx (v1.11) (|*r|* ≥ 0.5 and *P* < 0.05). In addition, using Spearman's correlation coefficients for exploratory the key volatile compounds and the top 10 genus (Xiao et al., 2015).

GC-IMS data were collected and analyzed using vocal software, including built-in Reporter and Gallery Plot plugins for plotting three-dimensional, two-dimensional, and fingerprint chromatograms of the volatile components (Chang et al., 2024). C4-C9 n-ketones were used as a reference to calculate the retention index (RI). The volatile components were identified using NIST 2020 (National Institute of Standards and Technology database), GC-IMS databases, and the RI index. The peak volume was used to calculate the relative quantities of the volatile compounds (Sun et al., 2023). A heatmap was generated using the online platform for data analysis and visualization available at https://www.bioinformatics.com.cn (last accessed on February 1, 2025). Multiple statistical analyses were performed using SIMCA (v14.1) for partial least squares discriminant analysis (PLS-DA) and variable importance in projection (VIP). SPSS software (v22.0) was used for the statistical analysis. One-way analysis of variance (ANOVA) and Duncan's multiple range test were used to assess the significance of the sample validity (*P* < 0.05). Spearman correlation coefficient heatmap was performed for the top ten genus and key substances using R (v3.3.1).

## 3 Results

### 3.1 Dynamic changes of VOCs in cigar tobacco leaf during mildew process analyzed by GC-IMS

**Figure 1:**
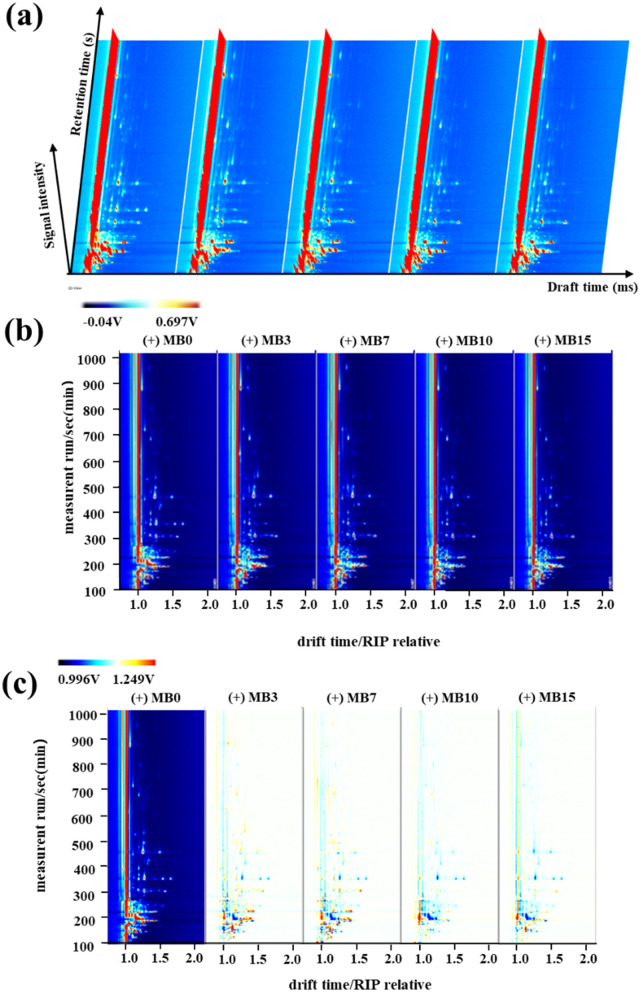
GC-IMS results of VOCs in cigar tobacco leaves at different samples. **(a)** Three-dimensional spectrum; **(b)** Two-dimensional spectrum; **(c)** Two-dimensional difference map.

GC-IMS is a type of separation and identification technology with strong separation ability and simple pretreatment process, which is widely used in food, herbs, wine, environment, and other fields (Cai et al., 2022; Chang et al., 2024; Chen et al., 2024; Christmann et al., 2024). This study employed GC-IMS to systematically investigate the spatiotemporal evolution of VOCs in cigar tobacco leaves during progressive mold development. The analytical outputs were visualized through three-dimensional, two-dimensional, and two-dimensional difference maps (Figure 1). In the three-dimensional representation (Figure 1a), vertical red peaks correspond to reaction ion peaks, flanked by discrete VOC signals whose color intensity reflects compound abundance-white indicating baseline levels and red denoting elevated concentrations (Zhao et al., 2024). Although GC-IMS demonstrated effective separation of cigar leaf VOCs, the three-dimensional visualization revealed limited capacity for inter-sample differentiation due to substantial qualitative similarities between mold stages. Enhanced resolution was achieved through two-dimensional spectral analysis (Figure 1b), where VOC profiles were color-coded by concentration gradients (red: high abundance; blue: low abundance). This representation facilitated comparative assessment of both quantitative and semi-quantitative variations across samples. To emphasize temporal changes during mold progression, differential spectral mapping was implemented (Figure 1c). The comparative analysis revealed distinct accumulation patterns, with MB3 and MB7 stages exhibiting elevated concentrations of specific VOCs that showed progressive attenuation in later stages (MB10 and MB15).

**Figure 2:**
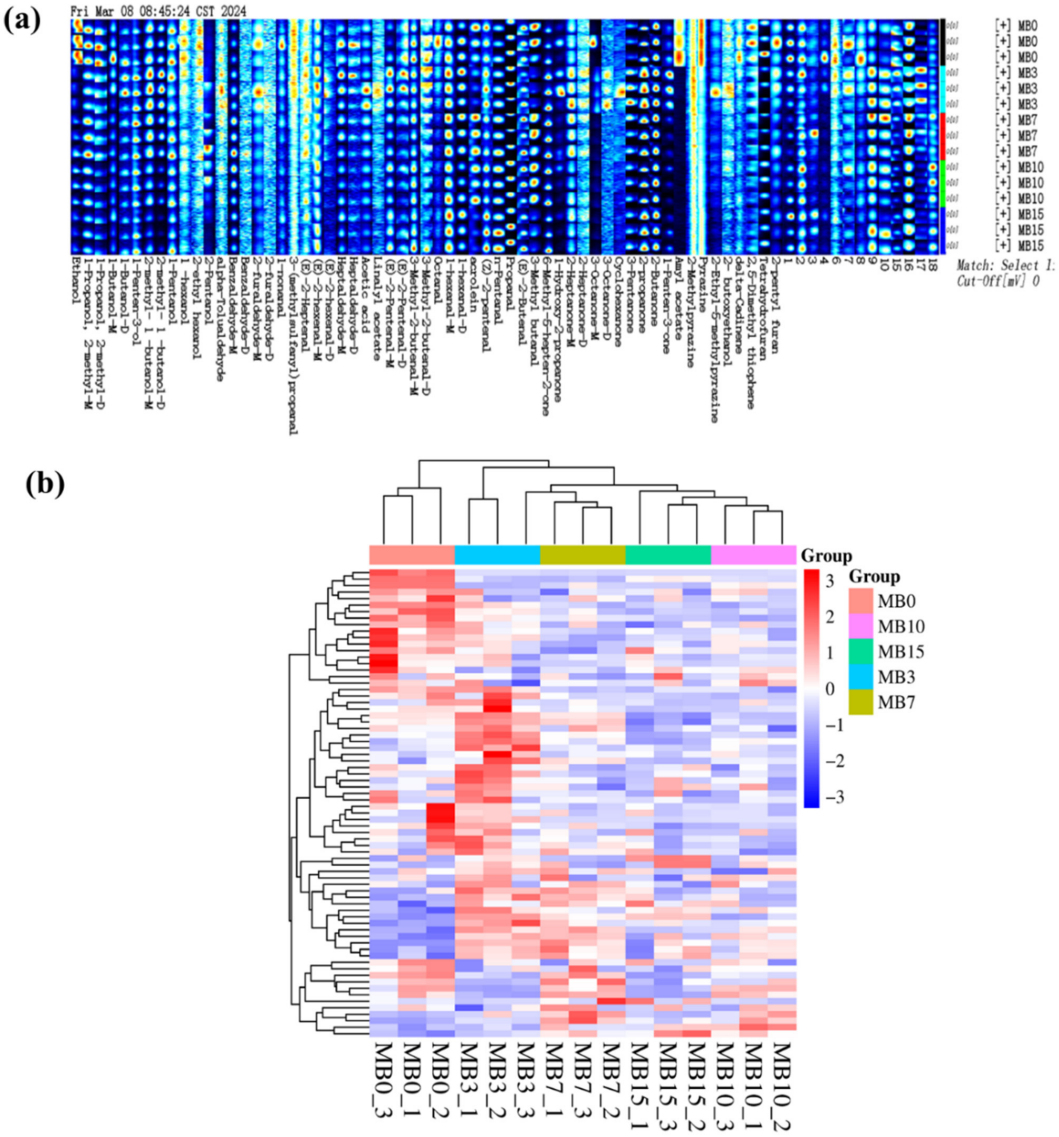
Fingerprint spectra **(a)** and heatmap **(b)** of VOCs in different samples.

Gallery Plot analysis resolved 72 VOCs across progression stages (Figure 2a), comprising 24 aldehydes (33.3%), 13 alcohols (18.1%), 12 ketones (16.7%), 1 organic acid, 2 esters, 3 pyrazines, 2 furans, 1 terpene, 1 thiophene, and 13 other substances (18.1%). Figure 2a illustrates a trend where the concentrations of ethanol, 1-propanol-2-methyl-D, amyl acetate, and pyrazine decreased subsequent to the onset of mold. Ethanol, a known product of microbial fermentation (Maicas, 2020), decreased in concentration, suggesting reduced microbial metabolic activity. Furthermore, studies have demonstrated a significant decrease in surface microbial diversity of tobacco leaves following mold contamination (Fu et al., 2024b; Wei et al., 2024; Zhang et al., 2024). 1-propanol-2-methyl-D, amyl acetate, and pyrazine have been reported as flavoring substances (Lakshmi et al., 2021; Müller and Rappert, 2010). The substantial decrease in these substances may signify a change in cigar leaf quality. The detailed information of VOCs is presented in Supplementary Table 1. Hierarchical clustering segregated samples into three contamination phases: Early (MB3), Middle (MB7), and Late (MB10, MB15), with MB0 forming a distinct control cluster (Euclidean). The heat map clustering clearly demonstrates substantial differences in VOC content at different mold durations, thus facilitating the potential for further monitoring of the early stages of tobacco molding (Figure 2b).

**Figure 3:**
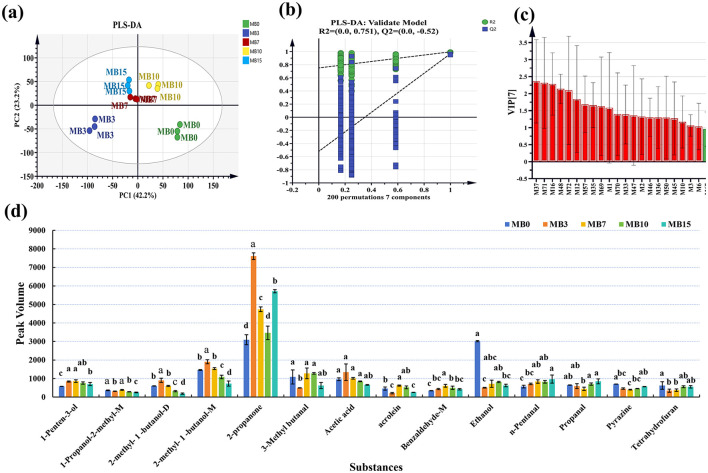
Statistical analysis of the VOCs in different groups. **(a)** PLS-DA; **(b)** Permutation test results (*n* = 200); **(c)** VIP score plot of the PLS-DA model; **(d)** Specific contents of 14 compounds with significant differences.

Partial least squares discriminant analysis (PLS-DA) demonstrated distinct clustering patterns among groups (Figure 3a). MB0 and MB3 exhibited significant separation from other groups, with MB3 representing the earliest detectable mold stage. To assess the model's performance metrics, 7-fold cross-validation and permutation tests (200 iterations) were employed in PLS-DA (Figure 3b). The PLS-DA score plot revealed the model's robust predictive capability (R2X = 0.91, R2Y = 0.975, Q2 = 0.776) (Figure 3b) (Westerhuis et al., 2008). The successful application of GC-IMS in classifying varying degrees of mold in peanuts and corn provides foundational support for the feasibility of using this technology to analyze VOCs associated with different levels of mold growth in tobacco leaves (Chen et al., 2021; Li et al., 2021b). Furthermore, 21 significantly different VOCs (VIP > 1), including M37, M71, M16, M48, M72, M12, M57, M35, M69, M1, M70, M33, M47, M2, M46, M36, M50, M45, M10, M3, and M6 were identified (Figure 3c). Among them, the VIP of M37 is the highest, with a 2.36. For a comprehensive list of all metabolites and their VIP scores, please refer to Supplementary Table 2. A total of 14 compounds were matched with compound names in the GC-IMS database. Following screening with criteria of *P* < 0.05 and VIP > 1 (Figure 3d), four compounds-−2-methyl-1-butanol-M, 2-methyl-1-butanol-D, 2-propanone, and 1-penten-3-ol—demonstrated elevated concentrations in MB3, positioning them as promising early-warning biomarkers for mold progression in cigar tobacco leaves. The specific content and variations in VOCs among different samples were shown in Table 1.

**Table 1:** Results of the analysis of the differences in the relative content of volatiles in the samples.

**No**.	**Name**	**MB0**	**MB3**	**MB7**	**MB10**	**MB15**
M1	2-methyl-1-butanol-M	0.6849 ± 0.0153^a^	0.8786 ± 0.0459^a^	0.7532 ± 0.0244^a^	0.5432 ± 0.0490^a^	0.3756 ± 0.0745^a^
M2	2-methyl-1-butanol-D	0.3186 ± 0.0065^ab^	0.4586 ± 0.0512^a^	0.3198 ± 0.0149^ab^	0.1721 ± 0.0254^bc^	0.0895 ± 0.0277^c^
M3	1-Penten-3-ol	0.4851 ± 0.0049^c^	0.7345 ± 0.0153^a^	0.7345 ± 0.0701^ab^	0.6474 ± 0.0555^abc^	0.5994 ± 0.1205^bc^
M4	1-Hexanol	0.0378 ± 0.0021^a^	0.0317 ± 0.0063^ab^	0.0273 ± 0.0046^b^	0.0326 ± 0.0039^ab^	0.0293 ± 0.0056^ab^
M5	1-Pentanol	0.2018 ± 0.0376^a^	0.1424 ± 0.0339^b^	0.1648 ± 0.0226^ab^	0.1518 ± 0.0119^ab^	0.1481 ± 0.0272^ab^
M6	2-Pentanol	0.0468 ± 0.0088^c^	0.1013 ± 0.0520^bc^	0.2104 ± 0.0582^ab^	0.1546 ± 0.0630^a^	0.0480 ± 0.0143^c^
M7	2-Butoxyethanol	0.0281 ± 0.0021^a^	0.0236 ± 0.0025^a^	0.0240 ± 0.0031^a^	0.0244 ± 0.0032^a^	0.0248 ± 0.0028^a^
M8	1-Butanol-M	0.3959 ± 0.1540^a^	0.2730 ± 0.1121^a^	0.2442 ± 0.0194^a^	0.2429 ± 0.0173^a^	0.2405 ± 0.0708^a^
M9	1-Butanol-D	0.0586 ± 0.0190^a^	0.0700 ± 0.0240^a^	0.0533 ± 0.0019^a^	0.0492 ± 0.0058^a^	0.0488 ± 0.0061^a^
M10	1-Propanol-2-methyl-M	0.4863 ± 0.0317^a^	0.3373 ± 0.0281^bc^	0.4692 ± 0.0434^a^	0.3658 ± 0.0131^b^	0.3044 ± 0.0180^c^
M11	1-Propanol-2-methyl-D	0.1961 ± 0.0268^a^	0.1335 ± 0.0099^b^	0.1241 ± 0.0131^bc^	0.0863 ± 0.0067^d^	0.1042 ± 0.0092^cd^
M12	Ethanol	1.4788 ± 0.0031^a^	0.4016 ± 0.0208^c^	0.6572 ± 0.1762^abc^	0.8619 ± 0.0381^ab^	0.4952 ± 0.0652^bc^
M13	2-Ethyl hexanol	0.0301 ± 0.0014^a^	0.0273 ± 0.0019^ab^	0.0248 ± 0.0028^b^	0.0256 ± 0.0021^b^	0.0236 ± 0.0031^b^
M14	Benzaldehyde-M	0.1176 ± 0.0063^c^	0.1461 ± 0.0039^bc^	0.2214 ± 0.0346^a^	0.1856 ± 0.0355^ab^	0.1538 ± 0.018b^c^
M15	Benzaldehyde-D	0.0228 ± 0.0019^a^	0.0195 ± 0.0024^a^	0.0212 ± 0.0039^a^	0.0208 ± 0.0000^a^	0.0195 ± 0.0024^a^
M16	3-Methyl butanal	1.6635 ± 0.5402^a^	0.6063 ± 0.0295^c^	1.6460 ± 0.2966^ab^	1.8124 ± 0.0917^ab^	0.7231 ± 0.1864^bc^
M17	2-Furaldehyde-M	0.0818 ± 0.0141^ab^	0.0863 ± 0.0257^a^	0.0598 ± 0.0053^bc^	0.0525 ± 0.0032^c^	0.0549 ± 0.0032^c^
M18	2-Furaldehyde-D	0.0183 ± 0.0012^ab^	0.0212 ± 0.0025^a^	0.0175 ± 0.0014^b^	0.0175 ± 0.0014^b^	0.0183 ± 0.0021^ab^
M19	Alpha-Tolualdehyde	0.0260 ± 0.0019^a^	0.0285 ± 0.0025^a^	0.0269 ± 0.0044^a^	0.0285 ± 0.0007^a^	0.0252 ± 0.0031^a^
M20	1-Nonanal	0.0574 ± 0.0268^a^	0.0509 ± 0.0072^a^	0.0439 ± 0.0097^a^	0.0399 ± 0.0049^a^	0.0444 ± 0.0104^a^
M21	3-(Methylsulfanyl) propanal	0.0407 ± 0.0014^a^	0.0407 ± 0.0025^a^	0.0338 ± 0.0007^b^	0.0334 ± 0.0025^b^	0.0346 ± 0.0014^b^
M22	(E)-2-Hexenal-M	0.1327 ± 0.0083^ab^	0.1603 ± 0.0132^a^	0.1261 ± 0.0184^b^	0.1384 ± 0.0223^ab^	0.1473 ± 0.0159^ab^
M23	3-Methyl-2-butenal-M	0.2975 ± 0.0415^a^	0.2665 ± 0.0205^a^	0.2515 ± 0.0331^a^	0.2840 ± 0.0197^a^	0.2804 ± 0.0577^a^
M24	3-Methyl-2-butenal-D	0.0501 ± 0.0085^a^	0.0537 ± 0.0088^a^	0.0427 ± 0.0012^a^	0.0411 ± 0.0037^a^	0.0423 ± 0.0132^a^
M25	Heptaldehyde-M	0.2389 ± 0.0683^ab^	0.2930 ± 0.0629^a^	0.2405 ± 0.0254^ab^	0.1884 ± 0.0189^b^	0.1998 ± 0.0366^b^
M26	Heptaldehyde-D	0.0411 ± 0.0155^a^	0.0435 ± 0.0129^a^	0.0321 ± 0.0046^a^	0.0265 ± 0.0046^a^	0.0273 ± 0.0056^a^
M27	(E)-2-Pentenal-M	0.1677 ± 0.0568^c^	0.3955 ± 0.0624^a^	0.2674 ± 0.0361^b^	0.3011 ± 0.0486^b^	0.3182 ± 0.0222^ab^
M28	(E)-2-Pentenal-D	0.0741 ± 0.0058^ab^	0.0903 ± 0.0211^a^	0.0570 ± 0.0143^b^	0.0541 ± 0.0140^b^	0.0671 ± 0.0112^ab^
M29	(E)-2-Heptenal	0.0354 ± 0.0037^a^	0.0411 ± 0.0070^a^	0.0415 ± 0.0032^a^	0.0391 ± 0.0056^a^	0.0346 ± 0.0037^a^
M30	(Z)-2-Pentenal	0.1636 ± 0.0265^a^	0.1823 ± 0.0069^a^	0.1542 ± 0.0031^a^	0.1685 ± 0.0146^a^	0.2153 ± 0.0046^a^
M31	(E)-2-Butenal	0.2185 ± 0.0192^a^	0.1823 ± 0.0425^a^	0.2397 ± 0.0479^a^	0.2197 ± 0.0429^a^	0.2128 ± 0.0335^a^
M32	1-Hexanal-M	0.9591 ± 0.0443^b^	1.0552 ± 0.0505^ab^	1.0731 ± 0.0342^a^	1.0149 ± 0.0600^ab^	1.0816 ± 0.0619^a^
M33	1-Hexanal-D	0.7850 ± 0.1722^ab^	0.9034 ± 0.1756^a^	0.8578 ± 0.0839^ab^	0.6104 ± 0.1096^b^	0.7634 ± 0.1626^ab^
M34	(E)-2-Hexenal-D	0.0305 ± 0.0012^b^	0.0525 ± 0.0056^a^	0.0334 ± 0.0007^b^	0.0244 ± 0.0032^c^	0.0203 ± 0.0025^c^
M35	n-Pentanal	1.1292 ± 0.1138^c^	1.2847 ± 0.0527^bc^	1.5227 ± 0.1425^ab^	1.5304 ± 0.1072^ab^	1.7351 ± 0.2893^a^
M36	Propanal	1.9781 ± 0.0789^a^	2.2369 ± 0.0846^a^	2.0334 ± 0.2625^a^	2.1144 ± 0.1619^a^	2.3276 ± 0.2989^a^
M37	Acrolein	1.2928 ± 0.3166^a^	0.5392 ± 0.1157^b^	1.5968 ± 0.0968^a^	1.4137 ± 0.2368^a^	0.5038 ± 0.0314^b^
M38	6-Methyl-5-hepten-2-one	0.2881 ± 0.0342^b^	0.4619 ± 0.0282^a^	0.4680 ± 0.0113^a^	0.4346 ± 0.0390^a^	0.3740 ± 0.0917^ab^
M39	1-Hydroxy-2-propanone	0.1139 ± 0.0903^a^	0.2144 ± 0.1441^a^	0.0806 ± 0.0307^a^	0.0834 ± 0.0552^a^	0.0529 ± 0.0242^a^
M40	2-Heptanone-M	0.0879 ± 0.0068^c^	0.1416 ± 0.0172^a^	0.1314 ± 0.0092^ab^	0.0985 ± 0.0173^c^	0.1070 ± 0.0185^bc^
M41	Octanal	0.0643 ± 0.0282^a^	0.0496 ± 0.0070^a^	0.0317 ± 0.0044^b^	0.0330 ± 0.0053^b^	0.0362 ± 0.0067^ab^
M42	2-Heptanone-D	0.0240 ± 0.0035^b^	0.0321 ± 0.0028^a^	0.0309 ± 0.0019^a^	0.0244 ± 0.0012^b^	0.0244 ± 0.0032^b^
M43	3-Octanone-D	0.0191 ± 0.0019^b^	0.0326 ± 0.0043^a^	0.0191 ± 0.0019^b^	0.0195 ± 0.0024^b^	0.0187 ± 0.0019^b^
M44	Cyclohexanone	0.0212 ± 0.0025^a^	0.0354 ± 0.0141^a^	0.0216 ± 0.0028^a^	0.0224 ± 0.0039^a^	0.0220 ± 0.0032^a^
M45	1-Penten-3-one	0.2018 ± 0.0515^b^	0.4680 ± 0.0908^a^	0.1278 ± 0.0322^b^	0.1359 ± 0.0518^b^	0.1815 ± 0.0559^b^
M46	3-Pentanone	0.4932 ± 0.0473^c^	1.0885 ± 0.1560^a^	0.8021 ± 0.1258^b^	0.7158 ± 0.0508^b^	0.7439 ± 0.0801^b^
M47	2-Butanone	3.8723 ± 0.1650^a^	4.0412 ± 0.0721^a^	4.0107 ± 0.1552^a^	3.7189 ± 0.3434^a^	3.8259 ± 0.1325^a^
M48	2-Propanone	4.5938 ± 0.1152^c^	5.7222 ± 0.0201^a^	5.2575 ± 0.0877^abc^	4.7439 ± 0.1814^bc^	5.4764 ± 0.0152^ab^
M49	3-Octanone-M	0.1742 ± 0.2393^a^	0.1913 ± 0.0151^a^	0.0529 ± 0.0007^a^	0.0680 ± 0.0014^a^	0.0557 ± 0.0087^a^
M50	Acetic acid	0.4358 ± 0.1408^a^	0.7878 ± 0.2577^a^	0.6079 ± 0.0324^a^	0.4969 ± 0.0160^a^	0.3715 ± 0.0128^a^
M51	Linalyl acetate	0.0224 ± 0.0007^ab^	0.0244 ± 0.0012^a^	0.0228 ± 0.0019^ab^	0.0203 ± 0.0019^b^	0.0208 ± 0.0012^b^
M52	Amyl acetate	0.1640 ± 0.0220^a^	0.0208 ± 0.0000^ab^	0.0171 ± 0.0021^b^	0.0199 ± 0.0031^ab^	0.0183 ± 0.0012^b^
M53	Pyrazine	0.2140 ± 0.0014^a^	0.1294 ± 0.0118^bc^	0.1205 ± 0.0056^c^	0.1359 ± 0.0031^abc^	0.1567 ± 0.0019^ab^
M54	2-Ethyl-5-methylpyrazine	0.0383 ± 0.0035^a^	0.0533 ± 0.0300^a^	0.0289 ± 0.0037^abc^	0.0256 ± 0.0032^bc^	0.0216 ± 0.0019^c^
M55	2-Methylpyrazine	0.0501 ± 0.0021^c^	0.0602 ± 0.0025^a^	0.0602 ± 0.0019^a^	0.0566 ± 0.0025^ab^	0.0529 ± 0.0007^bc^
M56	2-Pentyl furan	0.3548 ± 0.1307^a^	0.3272 ± 0.0614^a^	0.2543 ± 0.0536^ab^	0.1933 ± 0.0193^b^	0.1656 ± 0.0489^b^
M57	Tetrahydrofuran	1.7315 ± 0.5124^a^	0.9518 ± 0.2095^c^	1.1426 ± 0.1318^bc^	1.4446 ± 0.1270^ab^	1.4295 ± 0.1553^ab^
M58	Delta-Cadinene	0.0423 ± 0.0058^a^	0.0362 ± 0.0007^ab^	0.0281 ± 0.0021^c^	0.0317 ± 0.0044^bc^	0.0309 ± 0.0007^bc^
M59	2,5-Dimethyl thiophene	0.0956 ± 0.0164^a^	0.0671 ± 0.0024^ab^	0.0541 ± 0.0095^b^	0.0505 ± 0.0060^b^	0.0480 ± 0.0058^b^
M60	1	0.2214 ± 0.0309^a^	0.1481 ± 0.0250^bc^	0.0826 ± 0.0272^c^	0.1339 ± 0.0291^bc^	0.1799 ± 0.0593^ab^
M61	2	0.2564 ± 0.0204^a^	0.2165 ± 0.0672^a^	0.2340 ± 0.0070^a^	0.2714 ± 0.0264^a^	0.2352 ± 0.0314^a^
M62	3	0.0403 ± 0.0044^a^	0.0334 ± 0.0035^a^	0.0631 ± 0.0381^a^	0.0326 ± 0.0043^a^	0.0631 ± 0.0266^a^
M63	4	0.0932 ± 0.0705^a^	0.0509 ± 0.0266^a^	0.0277 ± 0.0035^a^	0.0330 ± 0.0032^a^	0.0378 ± 0.0160^a^
M64	6	0.0419 ± 0.0067^a^	0.0366 ± 0.0032^ab^	0.0317 ± 0.0012^b^	0.0321 ± 0.0025^b^	0.0330 ± 0.0021^b^
M65	7	0.0529 ± 0.0171^a^	0.0525 ± 0.0136^a^	0.0419 ± 0.0067^a^	0.0439 ± 0.0106^a^	0.0452 ± 0.0139^a^
M66	8	0.0708 ± 0.0203^a^	0.0545 ± 0.0095^ab^	0.0374 ± 0.0037^c^	0.0525 ± 0.0042^abc^	0.0427 ± 0.0076^bc^
M67	9	0.4444 ± 0.0839^b^	0.6901 ± 0.0427^a^	0.6702 ± 0.0794^a^	0.6234 ± 0.0639^ab^	0.6027 ± 0.1635^ab^
M68	10	0.0912 ± 0.0311^b^	0.2100 ± 0.0194^a^	0.2140 ± 0.0573^a^	0.1607 ± 0.0367^ab^	0.1534 ± 0.0822^ab^
M69	15	0.9030 ± 0.0177^a^	0.6812 ± 0.0970^b^	0.4216 ± 0.0750^c^	0.3841 ± 0.0674^c^	0.4403 ± 0.0287^c^
M70	16	1.2415 ± 0.0826^ab^	1.0560 ± 0.0149^c^	1.3250 ± 0.1294^a^	1.1597 ± 0.0994^abc^	1.0767 ± 0.0885^bc^
M71	17	0.6055 ± 0.1554^b^	1.2700 ± 0.0607^a^	0.7219 ± 0.1096^b^	0.7548 ± 0.0346^b^	0.4094 ± 0.0635^c^
M72	18	0.6918 ± 0.1287^c^	0.7284 ± 0.1154^bc^	0.9669 ± 0.2139^bc^	1.3677 ± 0.1945^a^	1.0401 ± 0.1839^b^

Data are presented as mean ± SD. Different letters indicate significant differences.

### 3.2 Changes in fungal community during moldy process of cigar tobacco leaves

#### 3.2.1 Fungal community alpha- and beta-diversity

**Figure 4:**
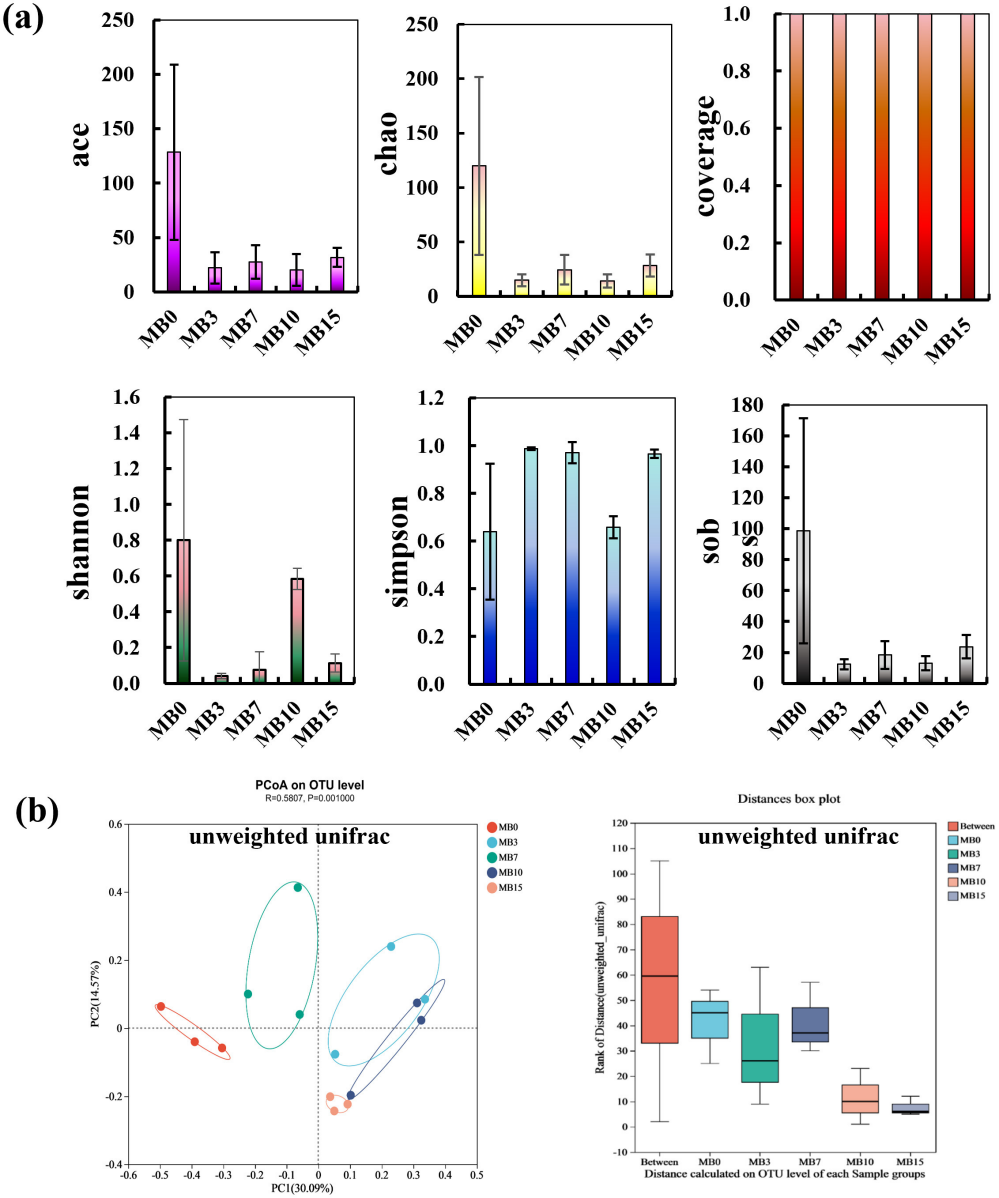
Fungal community diversity analysis across five groups. **(a)** Alpha diversity: ace, chao1, coverage, shannon, simpson, and sobs; **(b)** Beta diversity: PCoA based on unweighted Unifrac distances (axes explained 30.09% variance), with ANOSIM confirming significant inter-group differences (R = 0.5807, *P* = 0.001).

Alpha diversity indices (ace, chao1, sobs, and shannon) showed significant decrease following mold formation on cigar tobacco leaves (Figure 4a), indicating reduced fungal diversity and OTU richness. These observations align with prior reports of diminished mycobiota in mold-affected tobacco leaves (Fu et al., 2024b; Wei et al., 2024; Wu et al., 2024b; Zhou et al., 2024b). High sequencing coverage (99.93-99.99%) ensured reliable assessment of microbial diversity. Beta diversity analysis via unweighted UniFrac-based PCoA (Principal coordinates analysis) revealed distinct clustering patterns between moldy and control samples (Figure 4b). Progressive compositional shifts occurred across moldy stages, with ANOSIM confirming stronger inter-group than intra-group dissimilarities (R = 0.5807, *P* = 0.001). This statistically validated grouping underscores temporal dynamics in mold-associated mycobiota restructuring.

#### 3.2.2 Fungal OTU dynamics and annotation

**Figure 5:**
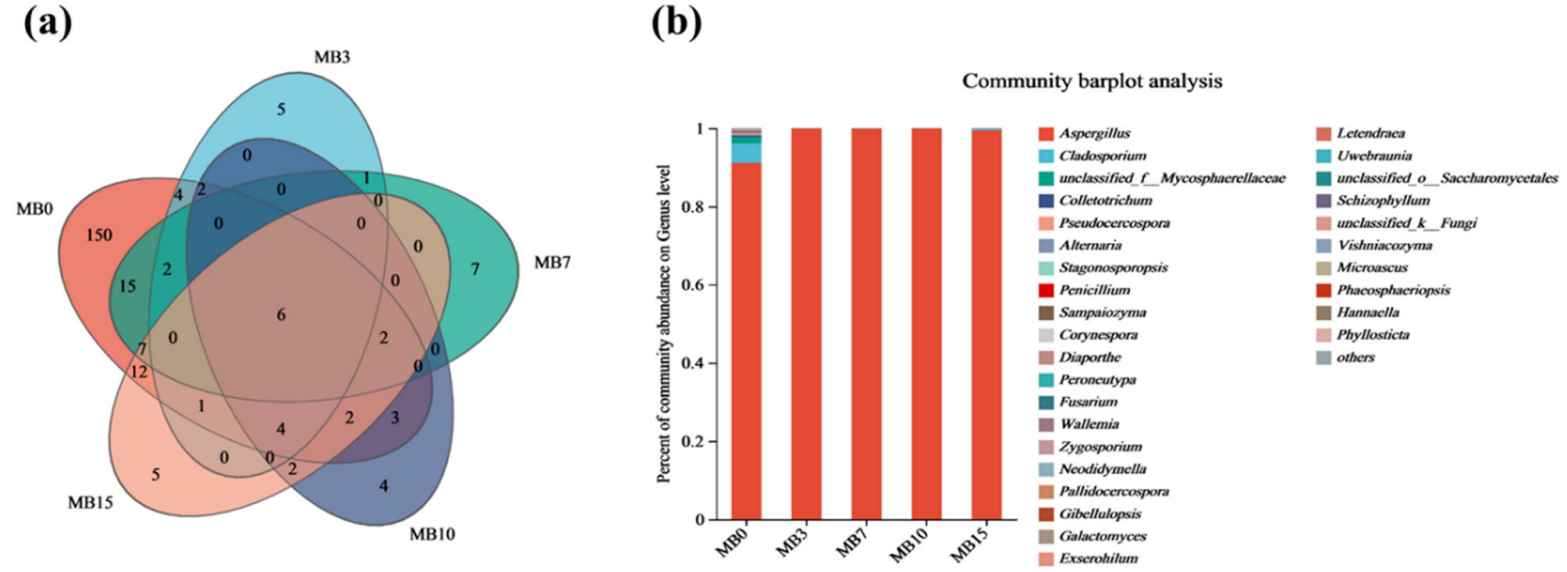
**(a)** Venn diagram of microbial OTU of fungi. **(b)** Species annotation at the genus level of fungal in five groups.

Venn analysis revealed drastic OTU reduction post-mold, with counts declining from 161 (MB0) to 21 (MB3), representing 85.7% species loss (Figure 5a). The OTU number of MB7, MB10, and MB15 is relatively stable, with 37, 22, and 34, respectively (Figure 5a). This collapse in fungal richness corroborates prior observations of mycobiota simplification in mold-degraded tobacco (Fu et al., 2024b; Wei et al., 2024). Genus-level profiling of the top 30 taxa demonstrated *Aspergillus* dominance, escalating from 91.15% (MB0) to > 99.45% in mold-affected samples (Supplementary Table 3 and Figure 5b). *Aspergillus* was a fungal species associated with a high percentage of moldy tobacco leaves (Wei et al., 2024; Wu et al., 2024a,b; Zhou et al., 2021a). Notably, *Cladosporium*—a putative pathogen (Feng et al., 2024)—constituted 4.88% of MB0 but collapsed to < 0.3% post-mold, suggesting niche exclusion by *Aspergillus*. Minor taxa (< 1% total abundance) including *Colletotrichum* and *Penicillium* showed similar suppression patterns.

#### 3.2.3 Network analysis and spearman correlation analysis between fungal communities and compounds

**Figure 6:**
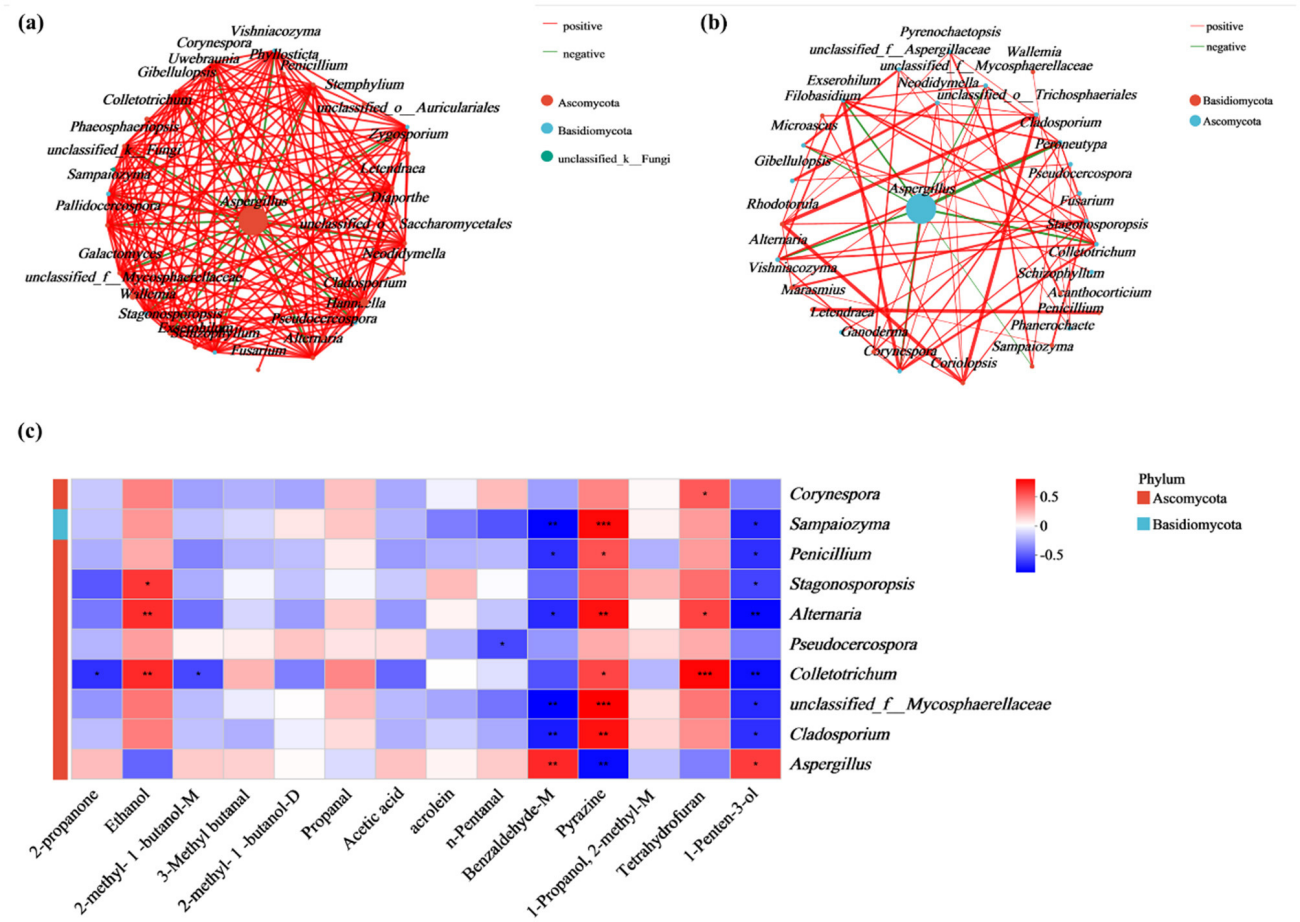
Fungal interaction networks and metabolite correlations. **(a)** Co-occurrence network of fungal communities in MB0 samples; **(b)** Network topology of molded samples (MB3, MB7, MB10, and MB15). The red line signifies a positive correlation between the two fungi, while the green indicates negative. Dots means the species of fungi, with size proportional to relative abundance. **(c)** Spearman correlation heatmap between the top 10 fungal and 14 differentially abundant metabolite. Color intensity reflects correlation strength (red: positive, blue: negative). Significance levels: *P < 0.05, **P < 0.01, ***P < 0.001.

To further explore the interactions among the fungal communities before and after molding, the correlations among the 30 most abundant fungal species in the control samples (MB0) and the molded samples (MB3, MB7, MB10, MB15) were examined using network analysis (Ramayo-Caldas et al., 2016) (Figure 6a). The red line indicated a positive correlation between the two fungi, and the green line indicated a negative relationship between the two fungi. The dots represented different fungi, and the size of the dots indicated how many species were present in the sample, with the larger the dots, the higher the percentage of species. There were more interactions between fungi in the normal sample (MB0), and a variety of fungi showed antagonistic relationships with *Aspergillus*, such as *Cladosporium, Alternaria, Sampaiozyma, Penicillium*, and *Stagonosporopsis*, suggesting niche overlap or metabolic inhibition (Figure 6a). However, with the appearance of mold, the number of interactions between fungi decreased, and the number of species showing antagonistic interactions with *Aspergillus* also decreased. The interactions between microorganisms on the surface of cigar tobacco leaves were weakened, especially the antagonistic species with *Aspergillus* (Figure 6b). This aligns with reports of weakened microbial competition under environmental stress (Wu et al., 2024b).

Spearman's correlations were observed between dominant fungi and 14 significantly different compounds (Figure 6c). *Aspergillus* showed strong positive associations with 1-penten-3-ol (ρ = 0.61) and benzaldehyde-M (ρ = 0.67), but negative correlations with pyrazine (ρ = −0.71). Conversely, *Aspergillus*-antagonistic taxa (e.g., *Alternaria, Penicillium, Cladosporium, Colletotrichum, Pseudocercospora*) displayed inverse trends.

## 4 Discussions

### 4.1 Early-warning biomarkers in cigar tobacco leaves

In this study, we used *t*-tests and PLS-DA to identify significantly different substances in the mold of cigar tobacco, particularly identifying four early-warning biomarkers in MB3. Studies related to early-warning biomarkers for mold have attracted attention from various industries. However, substantial discrepancies persist in the kind of early-warning biomarkers across studies. For example, Chen et al. (2021) employed GC-IMS to realize the determination of early mold in rice. Li et al. (2021a) investigated the VOCs of maize kernels at different molding process, suggesting the ethyl acetate-D, ethyl acetate-M, 3-hydroxybutan-2-one-D, methyl-5-hepten-2-one, and dimethyl disulfide were warning molecules at early stage of mold. Qin et al. (2024) also investigated maize VOCs at different mold times using GC-IMS and found that butan-2-one, ethyl acetate-D, benzaldehyde, and pentan-2-one could be used as signal molecules in the early stages. This phenomenon likely stems from the dependency of VOC diversity not only on microbial species but also on strain-specific growth environments and developmental stages (Wheatley, 2002; Mayrhofer et al., 2006; Misztal et al., 2018). Researches on mold biomarkers in tobacco has been reported. For example, Yu et al. (2023) demonstrated that 1-octene-3-alcohol, 1-pentanol, and pentanal as early markers of mold in cigar tobacco leaves following infection by two strains of fungi. Similarly, Wei et al. (2025) reported dynamic increases in characteristic volatiles during the molding process of both cigar wrapper and filler leaves. For example, 3-phenyl-2-propen-1-ol, cyclopentanone, 3-methyl-1-butanol, (Z)-3-hexenol, and 4-methoxybenzyl formate were characteristic volatile compounds in wrapper, and 1-pentanol-M, 3-methyl-1-butanol, 2-methyl-1-propanol-M, and 2-propenyl heptanoate were identified in filler. Some results have further expanded this field: Zheng et al. (2023) utilized SPME-GC-MS to screen eight mold-specific biomarkers in moldy tobacco, while Lin et al. (2023) employed GC-IMS to propose cis-3-hexen-1-ol, methyl butyrate, 2-pentanone, and ethylpropionic acid as potential marker for the moldy. The discrepancies between research findings may be attributed to the mold contamination levels, differential microbial metabolic activities, dissimilarities in tobacco leaf composition, or discrepancies in detection technologies. These findings underscore the necessity for subsequent studies to establish a standardized definition of early-stage mold contamination, identify predominant mold-causing species, and characterize their signature VOCs, which would significantly advance mold-related research in the tobacco industry. Furthermore, beyond VOC analysis, early-warning biomarkers for mold contamination could encompass monitoring alterations in mycotoxins and their precursors, dynamic changes in microbial populations and metabolic activities, as well as gene expression patterns and protein markers associated with mycotoxin biosynthesis pathways (Braissant et al., 2020; Fu et al., 2023, 2024a). Collectively, these findings provide a solid foundation for research on early warning systems for mold. The four early-warning biomarkers identified in this study were statistically validated through VIP > 1 and *P* < 0.05, however, their practical reliability under field conditions requires further verification. Subsequent investigations will systematically evaluate dose-response relationships between these biomarkers and mold severity, determine compound-specific threshold values, and establish operational protocols to guide industrial mold monitoring systems.

### 4.2 Correlation analysis between fungal communities and compounds

In essence, mildew formation results from the synergistic interaction among microbial communities, substrate properties, and environmental factors. Among these, the dynamic succession of fungal communities plays a critical role in identifying major fungal species. The characteristic of fungal community in molded samples was a drastic reduction in diversity and the overwhelming dominance of *Aspergillus* (>99.45%) in (Figure 5). This rapid ascendancy of *Aspergillus*, evident even in the early MB3 stage, aligns with previous research identifying it as a primary spoilage agent in tobacco (Fu et al., 2024b; Wei et al., 2024; Wu et al., 2024b; Zhou et al., 2024b). This underscores the critical importance of targeting *Aspergillus* in early mold prevention and control strategies.

Furthermore, correlation analyses between molds and substances provided insights into their interactions. The strong positive correlations established between *Aspergillus* and specific VOCs, 1-penten-3-ol (ρ = 0.61) and benzaldehyde-M (ρ = 0.67), strongly suggest that these compounds are significant metabolic compounds of *Aspergillus* activity on cigar tobacco. This direct linkage reinforces their utility as reliable indicators for early detection systems; an increase in these VOCs serves as a proxy for active *Aspergillus* proliferation. The association of 1-penten-3-ol with *Aspergillus* in this system is particularly noteworthy. While benzaldehyde is a recognized fungal volatile (Hung et al., 2015), and other typical “moldy” VOCs like 1-octen-3-ol are known. The prominent and early emergence of 1-penten-3-ol linked to *Aspergillus* in cigar tobacco suggests it could be a more specific or at least a highly sensitive early indicator in this particular substrate-microbe interaction.

Conversely, the significant negative correlation was observed between *Aspergillus* and pyrazine (ρ = −0.71). Pyrazines can be produced by various microorganisms and are sometimes associated with intrinsic tobacco aroma or early-stage microbial activity (Müller and Rappert, 2010). Its decline concomitant with *Aspergillus* proliferation likely reflects a multifaceted process: the suppression of other initial microbial colonizers by the aggressive growth of *Aspergillus*. This underscores that an early-warning signature may not solely rely on the appearance of new compounds but also on the significant alteration or disappearance of VOCs present in healthy or incipiently contaminated material.

## 5 Conclusions

In this study, GC-IMS was employed to analyze the VOCs of various moldy cigar tobacco leaves. A total of 72 VOCs were identified, among which 14 compounds exhibited significant differences (VIP > 1, *P* < 0.05). Specifically, 2-methyl-1-butanol-M, 2-methyl-1-butanol-D, 2-propanone, and 1-penten-3-ol were found to be at higher levels in the early—stage samples compared to others. These compounds could serve as early—warning biomarkers for tobacco mold. Furthermore, HTS results demonstrated that the number of species within the fungal communities decreased during the molding process of cigar tobacco leaves. *Aspergillus* was identified as the fungal species most closely associated with the molding process. Spearman's correlation analysis revealed that *Aspergillus* was significantly positively correlated with 1-penten-3-ol and benzaldehyde—M, while being significantly negatively correlated with pyrazine. Overall, this study achieved a remarkable feat by successfully uncovering the early-warning biomarkers for the early mold of cigar tobacco leaves. While these results are promising, there is considerable scope for further development. A key future direction will be the translation of these laboratory-validated biomarkers into practical, field-deployable sensor technologies for real-time, non-invasive monitoring in tobacco storage and processing facilities. Furthermore, future research also should aim to scale up the validation of the specificity and reliability of these biomarkers across a broader range of cigar tobacco varieties, curing processes, and diverse environmental conditions encountered in industrial settings. Conducting thorough industrial validation is crucial to support the integration of this approach into practical applications within the tobacco and relevant industries.

## Data Availability

The datasets presented in this study can be found in online repositories. The names of the repository/repositories and accession number(s) can be found below: https://www.ncbi.nlm.nih.gov/, PRJNA1215953.
