# Supplementary material for: GC-IMS identification of early-warning biomarkers and fungal community dynamics during cigar tobacco mold process
Source: Front Microbiol. 2025 Jul 16;16:1595849. doi: 10.3389/fmicb.2025.1595849 (PMC12307484; doi:10.3389/fmicb.2025.1595849)
Supplement: Supplementary file 1 [file Table_1.docx]

Supplementary Material

# Supplementary Tables

Supplement Table 1. Analysis of volatile organic compounds in cigar tobacco leaves by GC-IMS

|  | Compound | CAS | Formula | MW | RI | Rt [sec] | Dt [a.u.] | Comment |
| --- | --- | --- | --- | --- | --- | --- | --- | --- |
| Alcohols | 2-methyl-1-butanol-M | C137-32-6 | C5H12O | 88.1 | 1214.2 | 462.025 | 1.23576 | Monomer |
|  | 2-methyl-1-butanol-D | C137-32-6 | C5H12O | 88.1 | 1213.5 | 460.88 | 1.47486 | Dimer |
|  | 1-Penten-3-ol | C616-25-1 | C5H10O | 86.1 | 1165.4 | 389.76 | 0.94754 |  |
|  | 1-hexanol | C111-27-3 | C6H14O | 102.2 | 1366.8 | 712.705 | 1.32885 |  |
|  | 1-Pentanol | C71-41-0 | C5H12O | 88.1 | 1257.4 | 534.527 | 1.25143 |  |
|  | 2-butoxyethanol | C111-76-2 | C6H14O2 | 118.2 | 1402.6 | 772.338 | 1.20311 |  |
|  | 1-Butanol-M | C71-36-3 | C4H10O | 74.1 | 1170.4 | 396.777 | 1.17744 | Monomer |
|  | 2-Pentanol | C6032-29-7 | C5H12O | 88.1 | 1127.2 | 340.134 | 1.2088 |  |
|  | 1-Butanol-D | C71-36-3 | C4H10O | 74.1 | 1168.9 | 394.719 | 1.38573 | Dimer |
|  | 1-Propanol, 2-methyl-M | C78-83-1 | C4H10O | 74.1 | 1103.8 | 312.896 | 1.17399 | Monomer |
|  | 1-Propanol, 2-methyl-D | C78-83-1 | C4H10O | 74.1 | 1105.8 | 315.144 | 1.36274 | Dimer |
|  | Ethanol | C64-17-5 | C2H6O | 46.1 | 940.9 | 194.419 | 1.12505 |  |
|  | 2-Ethyl hexanol | C104-76-7 | C8H18O | 130.2 | 1495.1 | 949.956 | 1.40007 |  |
| Aldehydes | Benzaldehyde-M | C100-52-7 | C7H6O | 106.1 | 1501.5 | 963.672 | 1.1572 | Monomer |
|  | Benzaldehyde-D | C100-52-7 | C7H6O | 106.1 | 1500.3 | 961.102 | 1.4727 | Dimer |
|  | 2-furaldehyde-M | C98-01-1 | C5H4O2 | 96.1 | 1463.5 | 885.105 | 1.08811 | Monomer |
|  | alpha-Tolualdehyde | C122-78-1 | C_8_H_8_O | 120.2 | 1623.2 | 1265.622 | 1.25207 |  |
|  | 1-nonanal | C124-19-6 | C9H18O | 142.2 | 1398.2 | 764.744 | 1.47372 |  |
|  | 3-(methylsulfanyl)propanal | C3268-49-3 | C4H8OS | 104.2 | 1448.5 | 855.809 | 1.09183 |  |
|  | 2-furaldehyde-D | C98-01-1 | C5H4O2 | 96.1 | 1463 | 884.012 | 1.34446 | Dimer |
|  | (E)-2-hexenal-M | C6728-26-3 | C6H10O | 98.1 | 1222.5 | 475.189 | 1.17744 | Monomer |
|  | 3-Methyl-2-butenal-M | C107-86-8 | C5H8O | 84.1 | 1207.1 | 451.15 | 1.09346 | Monomer |
|  | 3-Methyl-2-butenal-D | C107-86-8 | C5H8O | 84.1 | 1207.5 | 451.722 | 1.36172 | Dimer |
|  | Heptaldehyde-M | C111-71-7 | C7H14O | 114.2 | 1190.2 | 425.966 | 1.32907 | Monomer |
|  | (E)-2-Pentenal-M | C1576-87-0 | C5H8O | 84.1 | 1140.5 | 356.712 | 1.11095 | Monomer |
|  | (E)-2-Pentenal-D | C1576-87-0 | C5H8O | 84.1 | 1139.2 | 354.995 | 1.36172 | Dimer |
|  | (E)-2-Heptenal | C18829-55-5 | C7H12O | 112.2 | 1333.4 | 661.402 | 1.25995 |  |
|  | (Z)-2-pentenal | C1576-86-9 | C5H8O | 84.1 | 1108.9 | 318.642 | 1.09566 |  |
|  | 1-hexanal-M | C66-25-1 | C6H12O | 100.2 | 1096.5 | 304.902 | 1.26644 | Monomer |
|  | 1-hexanal-D | C66-25-1 | C6H12O | 100.2 | 1096 | 304.402 | 1.56561 | Dimer |
|  | n-Pentanal | C110-62-3 | C5H10O | 86.1 | 998.8 | 224.766 | 1.4266 |  |
|  | Propanal | C123-38-6 | C3H6O | 58.1 | 795.5 | 135.623 | 1.15147 |  |
|  | Heptaldehyde-D | C111-71-7 | C7H14O | 114.2 | 1190.2 | 425.934 | 1.69821 | Dimer |
|  | (E)-2-hexenal-D | C6728-26-3 | C6H10O | 98.1 | 1225.9 | 480.67 | 1.51493 | Dimer |
|  | acrolein | C107-02-8 | C3H4O | 56.1 | 862 | 159.919 | 1.0625 |  |
|  | 3-Methyl butanal | C590-86-3 | C5H10O | 86.1 | 927.6 | 188.102 | 1.41281 |  |
|  | (E)-2-Butenal | C123-73-9 | C4H60 | 70.1 | 1060.4 | 272.427 | 1.20223 |  |
| Ketones- | 6-Methyl-5-hepten-2-one | C110-93-0 | C8H14O | 126.2 | 1347.1 | 682.066 | 1.18155 |  |
|  | 1-Hydroxy-2-propanone | C116-09-6 | C3H6O2 | 74.1 | 1311.2 | 629.338 | 1.22669 |  |
|  | 2-Heptanone-M | C110-43-0 | C7H14O | 114.2 | 1189.1 | 424.249 | 1.25675 | Monomer |
|  | Octanal | C124-13-0 | C8H16O | 128.2 | 1292.7 | 602.251 | 1.39788 |  |
|  | 2-Heptanone-D | C110-43-0 | C7H14O | 114.2 | 1186 | 419.505 | 1.62258 | Dimer |
|  | 3-Octanone-D | C106-68-3 | C8H16O | 128.2 | 1259.7 | 538.712 | 1.71858 | Dimer |
|  | Cyclohexanone | C108-94-1 | C6H10O | 98.1 | 1289.8 | 596.38 | 1.15326 |  |
|  | 1-Penten-3-one | C1629-58-9 | C5H8O | 84.1 | 1037.3 | 253.441 | 1.31138 |  |
|  | 3-Pentanone | C96-22-0 | C5H10O | 86.1 | 996.9 | 223.424 | 1.36063 |  |
|  | 2-Butanone | C78-93-3 | C4H8O | 72.1 | 916.9 | 183.17 | 1.23483 |  |
|  | 2-propanone | C67-64-1 | C3H6O | 58.1 | 831.5 | 148.257 | 1.1181 |  |
|  | 3-Octanone-M | C106-68-3 | C8H16O | 128.2 | 1262.6 | 544.015 | 1.29833 | Monomer |
| Acids | Acetic acid | C64-19-7 | C2H4O2 | 60.1 | 1462.2 | 882.553 | 1.05363 |  |
| Esters | Linalyl acetate | C115-95-7 | C12H20O2 | 196.3 | 1557.8 | 1093.078 | 1.22238 |  |
|  | Amyl acetate | C628-63-7 | C7H14O2 | 130.2 | 1180.8 | 411.795 | 1.30782 |  |
| Pyrazines | Pyrazine | C290-37-9 | C4H4N2 | 80.1 | 1223.2 | 476.253 | 1.05132 |  |
|  | 2-Ethyl-5-methylpyrazine | C13360-64-0 | C7H10N2 | 122.2 | 1387.3 | 746.313 | 1.16815 |  |
|  | 2-Methylpyrazine | C109-08-0 | C5H6N2 | 94.1 | 1269.9 | 557.607 | 1.08529 |  |
| Furans | 2-pentyl furan | C3777-69-3 | C9H14O | 138.2 | 1234.2 | 494.342 | 1.25014 |  |
|  | Tetrahydrofuran | C109-99-9 | C4H8O | 72.1 | 884.7 | 169.151 | 1.22931 |  |
| Terpene | delta-Cadinene | C483-76-1 | C15H24 | 204.4 | 1764.9 | 1738.608 | 1.46011 |  |
| Thiophene | 2,5-Dimethyl thiophene | C638-02-8 | C6H8S | 112.2 | 1159.6 | 381.804 | 1.07895 |  |
| Others | 1 | unidentified | * | 0 | 1254.1 | 528.586 | 1.12889 |  |
|  | 2 | unidentified | * | 0 | 1154.6 | 375.027 | 1.18094 |  |
|  | 3 | unidentified | * | 0 | 1152.9 | 372.738 | 1.21709 |  |
|  | 4 | unidentified | * | 0 | 1166.7 | 391.625 | 1.51685 |  |
|  | 6 | unidentified | * | 0 | 1244.8 | 512.391 | 1.32907 |  |
|  | 7 | unidentified | * | 0 | 1249.8 | 520.977 | 1.15411 |  |
|  | 8 | unidentified | * | 0 | 1342 | 674.249 | 1.30369 |  |
|  | 9 | unidentified | * | 0 | 1135.4 | 350.176 | 1.17708 |  |
|  | 10 | unidentified | * | 0 | 1137.2 | 352.506 | 1.41399 |  |
|  | 15 | unidentified | * | 0 | 961.6 | 204.639 | 1.19341 |  |
|  | 16 | unidentified | * | 0 | 922.8 | 185.854 | 1.32995 |  |
|  | 17 | unidentified | * | 0 | 936.9 | 192.476 | 1.37806 |  |
|  | 18 | unidentified | * | 0 | 1078.5 | 288.202 | 1.12088 |  |

Supplement Table 2. The VIP score of all volatile organic compounds

| No. | VIP score | No. | VIP score |
| --- | --- | --- | --- |
| M37 | 2.36101 | M25 | 0.619384 |
| M71 | 2.3139 | M31 | 0.580237 |
| M16 | 2.28178 | M30 | 0.576993 |
| M48 | 2.13973 | M63 | 0.552617 |
| M72 | 2.09711 | M23 | 0.527689 |
| M12 | 1.83869 | M40 | 0.487665 |
| M57 | 1.68285 | M59 | 0.482278 |
| M35 | 1.66002 | M61 | 0.4685 |
| M69 | 1.62707 | M5 | 0.466856 |
| M1 | 1.58284 | M66 | 0.450543 |
| M70 | 1.39465 | M22 | 0.437818 |
| M33 | 1.38514 | M17 | 0.41691 |
| M47 | 1.35942 | M28 | 0.389293 |
| M2 | 1.3228 | M41 | 0.386807 |
| M46 | 1.30294 | M34 | 0.365477 |
| M36 | 1.29708 | M54 | 0.359529 |
| M50 | 1.29582 | M9 | 0.322864 |
| M45 | 1.26951 | M43 | 0.279583 |
| M10 | 1.16642 | M20 | 0.278227 |
| M3 | 1.06291 | M58 | 0.269771 |
| M6 | 1.03481 | M24 | 0.263431 |
| M67 | 0.958031 | M26 | 0.260571 |
| M38 | 0.947667 | M65 | 0.245582 |
| M27 | 0.941728 | M44 | 0.241682 |
| M56 | 0.895085 | M55 | 0.236578 |
| M39 | 0.873201 | M4 | 0.22515 |
| M8 | 0.853183 | M42 | 0.218837 |
| M49 | 0.852871 | M21 | 0.215204 |
| M60 | 0.836702 | M64 | 0.211847 |
| M32 | 0.831916 | M29 | 0.210202 |
| M52 | 0.820833 | M13 | 0.166526 |
| M11 | 0.781199 | M19 | 0.160376 |
| M14 | 0.774067 | M51 | 0.150949 |
| M68 | 0.733258 | M7 | 0.121065 |
| M53 | 0.661305 | M18 | 0.120055 |
| M62 | 0.658304 | M15 | 0.0851608 |

Supplement Table 3. Statistics of the top 30 fungal species abundance in five samples

| OTU ID | MB0 | MB3 | MB7 | MB10 | MB15 |
| --- | --- | --- | --- | --- | --- |
| *Aspergillus* | 0.911498 | 0.999283 | 0.998704 | 0.999510 | 0.994488 |
| *Cladosporium* | 0.048770 | 0.000530 | 0.000630 | 0.000237 | 0.003051 |
| *unclassified_f__Mycosphaerellaceae* | 0.016568 | 0.000010 | / | / | 0.000080 |
| *Colletotrichum* | 0.005442 | / | / | 0.000030 | 0.000040 |
| *Pseudocercospora* | 0.002567 | 0.000015 | / | 0.000015 | / |
| *Alternaria* | 0.001947 | / | 0.000030 | 0.000005 | 0.000489 |
| *Stagonosporopsis* | 0.002300 | / | 0.000066 | 0.000010 | 0.000020 |
| *Penicillium* | 0.001700 | 0.000030 | 0.000010 | 0.000040 | 0.000388 |
| *Sampaiozyma* | 0.001785 | 0.000050 | 0.000030 | 0.000025 | 0.000136 |
| *Corynespora* | 0.000635 | / | 0.000091 | / | 0.000348 |
| *Diaporthe* | 0.001069 | / | / | / | / |
| *Peroneutypa* | 0.000010 | / | / | / | 0.000600 |
| *Fusarium* | 0.000580 | / | 0.000005 | / | 0.000005 |
| *Wallemia* | 0.000464 | 0.000035 | / | 0.000010 | 0.000005 |
| *Zygosporium* | 0.000464 | / | / | / | 0.000005 |
| *Neodidymella* | 0.000287 | / | 0.000131 | / | 0.000025 |
| *Pallidocercospora* | 0.000424 | / | / | / | / |
| *Gibellulopsis* | 0.000111 | / | 0.000040 | / | 0.000187 |
| *Galactomyces* | 0.000287 | / | / | / | / |
| *Exserohilum* | 0.000212 | / | 0.000010 | / | 0.000040 |
| *Letendraea* | 0.000222 | 0.000015 | / | / | / |
| *Uwebraunia* | 0.000166 | / | 0.000005 | / | / |
| *unclassified_o__Saccharomycetales* | 0.000151 | / | 0.000010 | / | / |
| *Schizophyllum* | 0.000111 | / | 0.000030 | / | / |
| *unclassified_k__Fungi* | 0.000136 | / | / | / | / |
| *Vishniacozyma* | 0.000101 | 0.000005 | 0.000010 | / | 0.000005 |
| *Microascus* | / | / | / | 0.000101 | 0.000020 |
| *Phaeosphaeriopsis* | 0.000116 | / | / | / | / |
| *Hannaella* | 0.000111 | / | 0.000005 | / | / |
| *Phyllosticta* | 0.000106 | / | / | / | / |
| *others* | 0.001659 | 0.000025 | 0.000192 | 0.000015 | 0.000066 |

Note: “/” means “zero”
